# The multimodal Ganzfeld-induced altered state of consciousness induces decreased thalamo-cortical coupling

**DOI:** 10.1038/s41598-020-75019-3

**Published:** 2020-10-29

**Authors:** Timo Torsten Schmidt, Nisha Jagannathan, Michal Ljubljanac, Ann Xavier, Till Nierhaus

**Affiliations:** 1 Neurocomputation and Neuroimaging Unit (NNU), Department of Education and Psychology, Freie Universität Berlin, Habelschwerdter Allee 45, 14195 Berlin, Germany; 2 Institute of Cognitive Science, University of Osnabrück, 49076 Osnabrück, Germany; 3 Department of Neurology, Max Planck Institute for Human Cognitive and Brain Sciences, 04103 Leipzig, Germany

**Keywords:** Cognitive neuroscience, Neural circuits

## Abstract

Different pharmacologic agents have been used to investigate the neuronal underpinnings of alterations in consciousness states, such as psychedelic substances. Special attention has been drawn to the role of thalamic filtering of cortical input. Here, we investigate the neuronal mechanisms underlying an altered state of consciousness (ASC) induced by a non-pharmacological procedure. During fMRI scanning, N = 19 human participants were exposed to multimodal Ganzfeld stimulation, a technique of perceptual deprivation where participants are exposed to intense, unstructured, homogenous visual and auditory stimulation. Compared to pre- and post-resting-state scans, the Ganzfeld data displayed a progressive decoupling of the thalamus from the cortex. Furthermore, the Ganzfeld-induced ASC was characterized by increased eigenvector centrality in core regions of the default mode network (DMN). Together, these findings can be interpreted as an imbalance of sensory bottom-up signaling and internally-generated top-down signaling. This imbalance is antithetical to psychedelic-induced ASCs, where increased thalamo-cortical coupling and reduced DMN activity were observed.

## Introduction

The induction of short-lasting altered states of consciousness (ASCs) are used as an experimental approach to study abnormal mental processing, which can occur in diverse mental diseases such as schizophrenia^1,2^. Recently, ASCs have been induced within neurophysiological experiments by the administration of psychedelic substances^3–7^. These ASCs share some phenomenological features with psychotic states, such as the emergence of multimodal (pseudo-) hallucinations^1,8,9^. However, no available substance has been able to induce and model all features of psychotic states^1,10–12^. Nevertheless, such experiments comprise a versatile research tool to elucidate relationships between altered brain processes and specific disturbances in perception and thinking. In particular, the temporary induction of ASCs allows for the testing of hypotheses of specific neuronal mechanisms in healthy participants, whose brains did not undergo long-term compensatory mechanisms as in most pathologies.

Given the complexity of biochemical interactions, the experimental use of pharmacological substances comes with obvious experimental confounds. Therefore, the comparison with non-pharmacological methods that induce ASCs is particularly informative. Here, we used the multimodal Ganzfeld (MMGF) technique to induce an ASC. In MMGF, participants are deprived of structure in their sensory inputs by high-intensity exposure to homogenous visual and auditory stimulation. The technique was coined by Wolfgang Metzger in the context of Gestalt psychology in the 1930s^13,14^ and has been optimized to create an unstructured sensory environment^13,15–19^. MMGF is referred to as a technique of *perceptual deprivation* that is contrary to *sensory deprivation,* as strong sensory stimulation is used that lacks structures to be perceptually processed. A few minutes of MMGF exposure typically induces an absorptive state, characterized by inward-directed thoughts and reduced vigilance, which is sometimes described as feeling like an eyes-open, elongated transition state between wakefulness and sleep, including effects on time perception^17,20^. Participants also commonly report vivid hallucinations with various auditory and visual contents, such as hearing sounds of water, voices, or traffic^13^. MMGF thereby comprises an elegant paradigm to experimentally test for neuronal mechanisms that contribute to ASC experiences.

Altered processing in cortico-striato-thalamo-cortical feedback loops are thought to crucially contribute to the emergence of ASC experiences. They are relevant for gating and filtering information transmitted from the sensory organs to the cortex. This filter function of the thalamus is thought to be impaired in schizophrenia and temporarily disrupted during pharmacologically-induced ASCs with psychedelics^7,8,21^. Studies from different research groups have replicated the finding that serotonergic psychedelics, such as LSD and psilocybin, lead to increased thalamo-cortical coupling, which is thought to reflect the unfiltered propagation of sensory information to the cortex^22^. This mechanism, also seen in schizophrenia and referred to as *sensory flooding*, has been suggested to be a driving factor for hallucinations^21,22^. On the contrary, trance states, which are experienced as inward-directed and as dissociating from the outside world, were found to induce a reduction in thalamo-cortical coupling^23^.

In the present study, we aimed to investigate how MMGF affects brain network interactions, including thalamo-cortical coupling. To this end, we constructed an fMRI-compatible MMGF setup for the dispersion of a uniform light source onto the eyes in conjunction with the presentation of auditory white noise. Participants were exposed to MMGF for 25 min during resting-state fMRI scanning, and we compared the MMGF-induced state to resting-state scans before and after. The induced MMGF-phenomenology was evaluated with retrospective questionnaires that enabled a direct comparison of MMGF and drug-induced states^9^. We hypothesized that MMGF exposure would have an effect on thalamo-cortical interactions and investigated these as changes in functional connectivity assessed as correlations between brain regions. We further tested for global connectivity changes by employing eigenvector centrality mapping.

## Methods

### Participants

Nineteen native German speakers with no history of psychiatric or neurological disorders participated in the experiment (13 female; 6 male; age range 19–35 years, *M* = 25.5, *SD* = 4.7). According to the Edinburgh Handedness Inventory^24^ all participants were right-handed (Mean laterality quotient = 90.1). Prior to the experiment, written informed consent was obtained from each participant. The study was approved by the ethics committee at the Freie Universität Berlin. All procedures were consistent with the guidelines included in the “Declaration of Helsinki—Ethical Principles for Medical Research Involving Human Subjects”.

### Multimodal Ganzfeld (MMGF) setup

**Figure 1:**
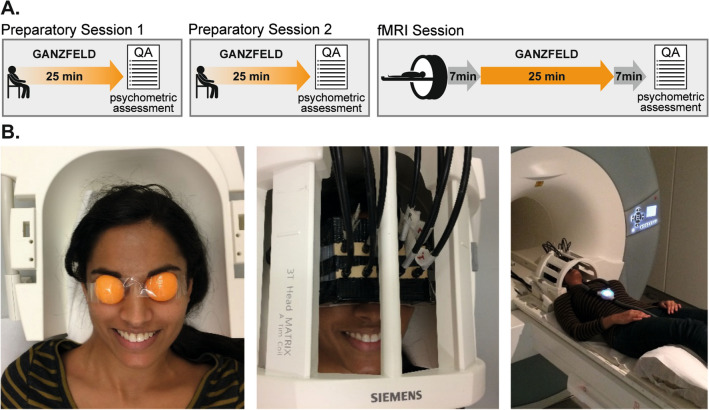
Experimental setup and study design. (**A**) Two preparatory sessions and one fMRI session were conducted on three different days. (**B**) fMRI compatible MMGF setup. Orange ping-pong balls were cut into halves and shaped to fit securely over the participant`s eye orbits. Homogenous light exposure was realized via strong LEDs and fiber-optic cables connected to custom made goggles, ensuring that no inhomogeneity or shadows from the headcoil would employ structure to the visual field. Auditory white noise was presented via MR-compatible headphones (The image shows one of the authors, who confirms consent for publication).

Visual homogeneity was achieved using a previously described and evaluated technique^13,16,25^. Orange ping-pong balls were cut into halves and shaped to fit securely over the participants’ eye orbits. The ping-pong ball halves were secured with transparent tape and a custom-built case was affixed over the participants’ eyes, mounting eight fiber-optic cables for translucent illumination of the eyes (Fig. 1B). The light was generated using eight white light-emitting diodes (LEDs), powered via a high-fidelity amplifier and a custom-built circuit of resistors. Since prior research suggested that warm colors, such as red, facilitate better immersion into the Ganzfeld^25^, orange ping-pong balls and white light were used to provide a warm orange tint to the visual field. Auditory homogeneity was achieved by exposing the participants to broad-band flat-spectrum white noise played through headphones at the loudest intensity comfortable for the participant (*M* = 79.8 dB, *SD* = 11.0 dB). Participants experienced the visual and auditory homogenization simultaneously for 25 min while lying in a horizontal relaxed position with eyes open.

### Experimental procedure

In order to familiarize participants with the MMGF setup and procedure, they were asked to complete two 25-min preparation sessions before participating in the resting-state fMRI session. The two preparation sessions and the fMRI scanning session were conducted on three separate days within the same week.

Prior to scanning, participants were instructed to insert a pair of earplugs before placing the headphones over their ears. The volume of the white noise was then personalized for each participant, ensuring that the scanner noise was barely audible during the MMGF condition. The scanning session consisted of a seven-minute, eyes-open, task-free resting-state scan, followed by a 25-min Ganzfeld functional scan. Following the Ganzfeld, an anatomical scan using a T1-weighted 3D-MPRAGE sequence was performed. Finally, a second seven-minute, eyes-open, task-free scan concluded the session (Fig. 1A). Participants were briefly moved out of the scanner twice during the scanning session: once to put on the MMGF setup (after the first seven-minute scan) and again to remove the MMGF setup (after the MMGF scan).

Phenomenological aspects of the Ganzfeld-induced state were retrospectively assessed using the Altered States of Consciousness Rating Scale^26,27^ which participants completed immediately after each MMGF session. This questionnaire has been successfully evaluated for validity and reliability, as well as highlighted as one of the most suitable psychometric tools for ASC research^9,28^.

### MRI data acquisition

Participants were scanned at the Center for Cognitive Neuroscience Berlin (CCNB), using a 3T Siemens Tim Trio MRI scanner equipped with a 12-channel head coil (Siemens Medical, Erlangen, Germany). For resting-state fMRI images a T2*-weighted echo planar imaging (EPI) sequence was used (37 axial slices acquired interleaved, in-plane resolution is 3 × 3 mm, slice thickness = 3 mm, flip angle = 70°, 20% gap, TR = 2000 ms, TE = 30 ms). A structural image was acquired for each participant using a T1-weighted MPRAGE sequence (TR = 1900 ms, inversion time = 900 ms, TE = 2.52 ms, flip angle = 9°, voxel size 1 × 1 × 1 mm). Participants’ heads were immobilized by cushioned supports.

### MRI data preprocessing

Data were preprocessed and analyzed in Matlab (version 9.5, The MathWorks, Natick, U.S.A.) using SPM12 (www.fil.ion.ucl.ac.uk/spm/) and a custom-built resting-state data analysis pipeline. The anatomical T1-images were normalized to MNI152 space using the segmentation approach, thus estimating a nonlinear transformation field which can also be used for normalization of the resting-state scans. The first three volumes of each resting-state scan were removed to account for adaptation of the participant to scanner noise and environment. We performed slice time correction, head motion correction, and spatial normalization to MNI152 space. The frame-wise displacement (FD) was calculated for each scan using BRAMILA tools^29^. Volumes that exceeded a threshold of 0.4 mm were replaced with an interpolation of the two neighboring volumes and were masked during following analysis steps (“*scrubbing*”). Furthermore, FD values were used to compare differences in head motion across resting-state scans. A principal component analysis (CompCor) was done using the DPABI toolbox (toolbox for Data Processing & Analysis of Brain Imaging, https://rfmri.org/dpabi) within the CSF/white matter mask on the resting-state data^30^ to estimate nuisance signals. Anatomical masks for CSF, white and grey matter were derived from the tissue-probability maps provided in SPM12. Smoothing was performed using a 6 mm FWHM Gaussian kernel. The first five principal components of the CompCor analysis, the six head motion parameters, as well as linear and quadratic trends were used as nuisance signals to regress out associated variance. Finally, the toolbox REST (www.restfmri.net) was used for temporal band-pass filtering (0.01–0.08 Hz).

### ROI to ROI correlation analysis

**Figure 2:**
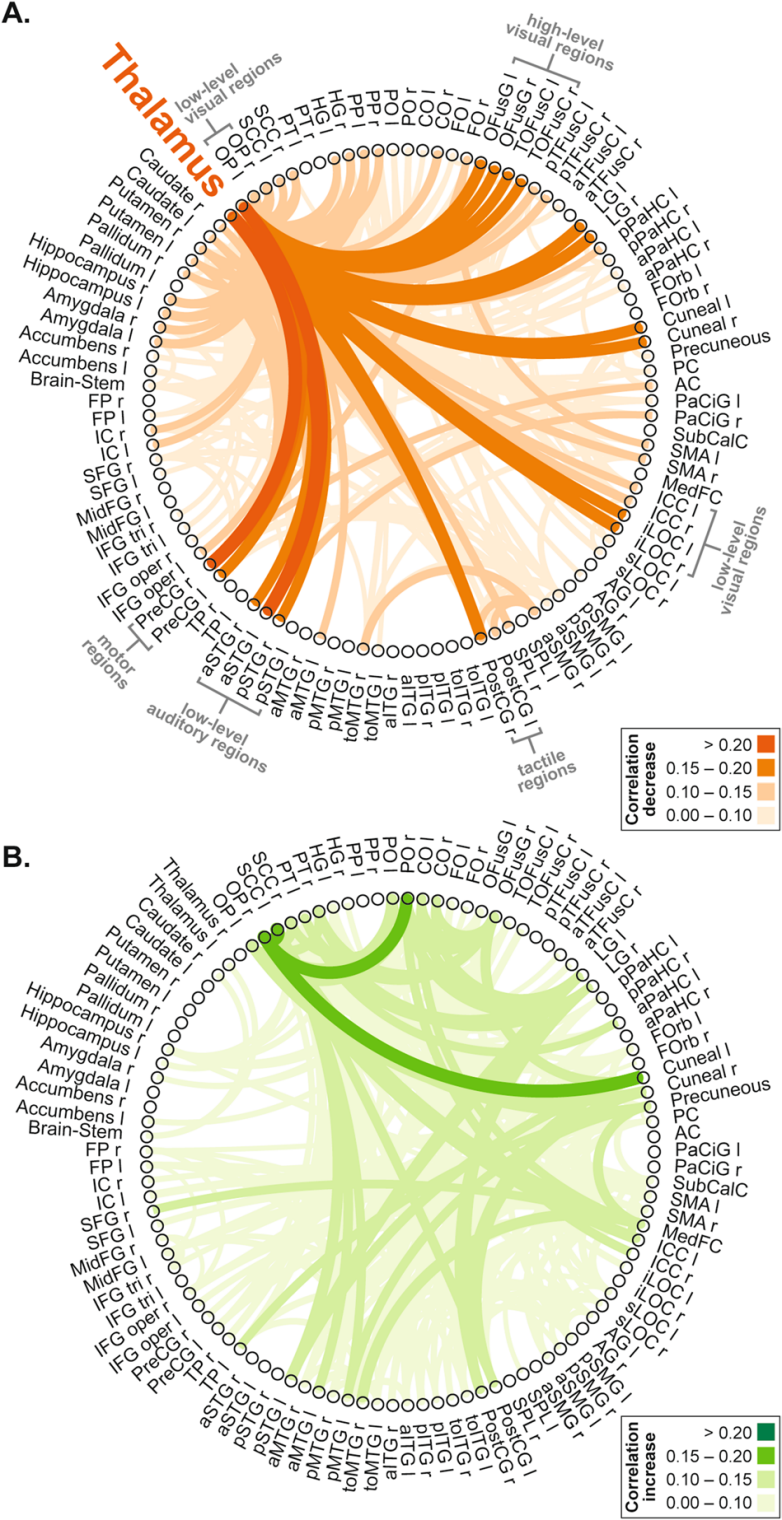
Changes in resting-state functional connectivity during MMGF. Decreases (**A**) and increases (**B**) in ROI-to-ROI temporal correlations for the comparison of MMGF to averaged pre and post scans. ROIs were defined according to the AAL atlas.

We used the automated anatomical labeling (AAL) atlas^31^ for definition of anatomical regions of interest (ROIs) of the whole brain. First, we extracted the mean BOLD time course from the 106 AAL regions of cortical and subcortical areas, excluding the cerebellum. To test for changes in the coupling of brain regions that characterize the MMGF-induced altered state of consciousness, we calculated the temporal correlation of BOLD time series between all ROIs. For all ROI-to-ROI pairs we averaged the correlation coefficients of pre and post scans and computed the difference to the MMGF scan. This corresponded to a subtraction of the connectivity matrices. To explore the pattern of Ganzfeld-induced connectivity changes, we plotted all decreases and increases of correlation coefficients (Fig. 2).

To directly test the hypothesis that thalamo-cortical coupling comprises a major mechanism of the Ganzfeld induced ASC, we statistically assessed the correlation coefficients between the thalamus and four selected cortical sensory areas. Therefore, we used the right and left thalamus (labelled in AAL with 92 and 93, respectively), the primary auditory cortex (A1) as anatomically defined by the posterior division of the right and left superior temporal gyrus (STG, labelled with 19 and 20, respectively), and the primary visual cortex (V1) as anatomically defined by the right and left intracalcarine cortex (ICC, labelled with 47 and 48, respectively). We tested the MMGF condition versus averaged pre and post scans and calculated one paired t-test to compare the correlation coefficients (average left and right hemisphere) between the thalamus and V1, as well as one paired t-test for the comparison between thalamus and A1. To further investigate the temporal development of functional connectivity during the MMGF scan, we divided the 25-min scan into five 5-min windows and calculated the correlation coefficients for each window.

To further explore whether different thalamic subregions showed different connectivity profiles, we used the thalamus parcellation provided in the AAL3 atlas^32^. It should be noted that this was performed as a post-hoc explorative analysis, without statistical assessment, with the purpose of displaying which thalamic nuclei and what cortical regions showed the strongest decoupling.

### Eigenvector centrality mapping

We used the data-driven eigenvector centrality mapping approach to characterize whole-brain functional connectivity without prior assumptions. This graph theoretical network approach quantifies the correlation of each voxel with all other voxels in the brain, aiming to identify how “central” (or prominent) this region is within the whole-brain network^33^. For each individual resting-state scan, the eigenvector centrality map has been generated within the grey matter mask by using fastECM^34^. For our statistical analysis we compared the eigenvector centrality maps during MMGF exposure to the averaged pre and post scans. This contrast was computed in a second-level ANOVA design in SPM12.

## Results

### MMGF-induced altered states of consciousness phenomenology

The retrospective assessment of the induced ASC phenomena using questionnaires demonstrated that the MMGF-induced state during the fMRI resting-state measurement was in line with previous reports about its phenomenology^13^. The 5D-ASC analysis scheme of the Altered States of Consciousness Rating Scale revealed Oceanic Boundlessness: 11.3 ± 13.2 [mean ± SEM]; Dread of Ego Dissolution: 6.7 ± 5.0; Visionary Restructuralization: 11.9 ± 14.5; Auditory Alterations: 11.9 ± 14.6; Vigilance Reduction: 29.1 ± 20.6. The 11-ASC analysis scheme revealed Anxiety: 2.6 ± 5.1; Auditory-visual synesthesia: 8.3 ± 10.4; Blissful state: 14.3 ± 19.1; Changed meaning of percepts: 5.9 ± 11.6; Complex imagery: 16.9 ± 25.5; Disembodiment: 13.2 ± 18.2; Elementary imagery: 17.9 ± 26.0; Experience of unity: 10.1 ± 15.8; Impaired control and cognition: 10.2 ± 9.1; Insightfulness: 11.4 ± 26.2; Spiritual experiences: 3.9. ± 5.7. We did not find a significant (*p* > 0.05) difference in any of these scores when testing for an effect of setting (i.e. inside vs. outside the MRI scanner), by testing with two-sided paired one-sample t-tests for a difference between the second preparatory session and the fMRI session.

### Control analysis: motion comparison between scans

Motion did not significantly differ between pre, MMGF, and post scans as assessed by (1) mean frame-wise displacement (FD; pre: 0.14 ± 0.05 mm [mean ± std]; MMGF: 0.14 ± 0.05 mm; post: 0.15 ± 0.05 mm; 1 × 3 repeated-measures ANOVA: n.s.) and (2) percentage of volumes exceeding an FD-threshold of 0.4 (pre: 1.9 ± 2.9% [mean ± std]; MMGF: 2.2 ± 2.6%; post: 3.1 ± 5.0%; 1 × 3 repeated-measures ANOVA: n.s.).

### MMGF-induced changes in thalamo-cortical functional connectivity

All changes in ROI-to-ROI correlation coefficients between MMGF and averaged pre and post scans are displayed in Fig. 2. In this explorative depiction, decreases in coupling of the thalamus to sensory regions were predominantly apparent (Fig. 2A). Furthermore, we found a significantly decreased correlation when comparing the correlation coefficients (average left and right hemisphere) between the thalamus and V1 (paired t-test, *t*(18) = 2.67, *p* = 0.016). Testing the correlation decrease between thalamus and A1 with a paired t-test revealed *t*(18) = 2.09, *p* = 0.051.

**Figure 3:**
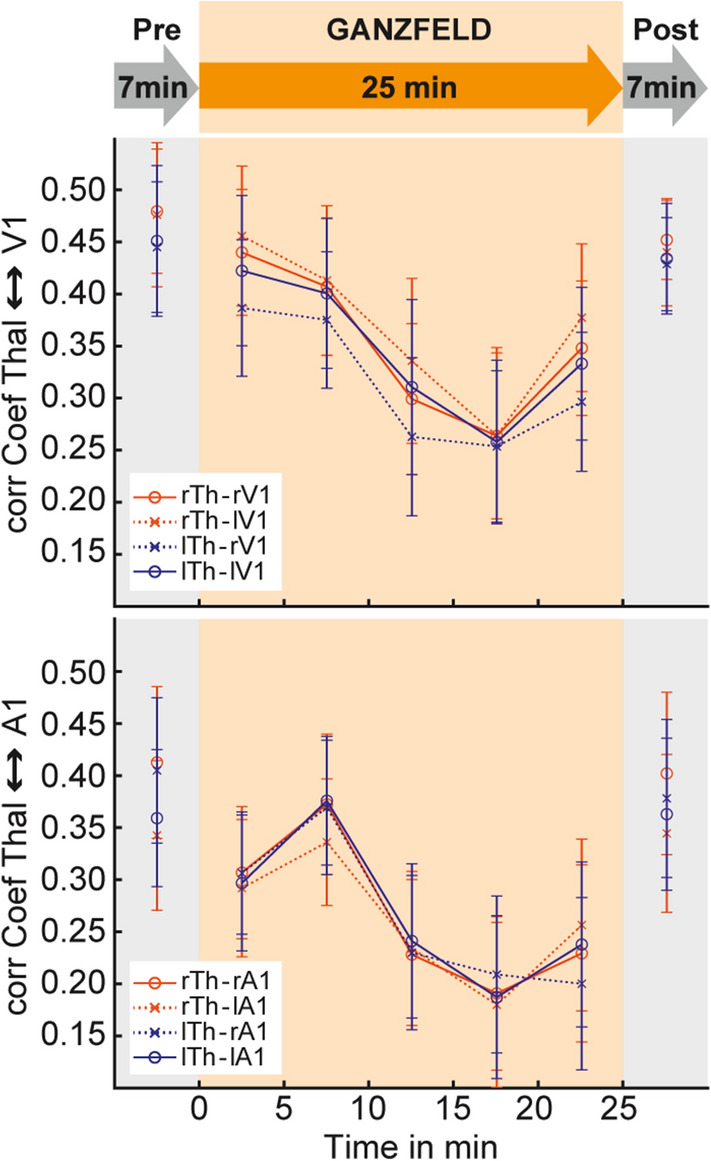
Time-resolved resting-state analysis of thalamo-cortical interactions. A progressive decrease in correlation between the thalamus and V1 as well as the thalamus and A1 across the MMGF exposure reflects a cortical decoupling from sensory thalamic inputs.

The temporal development in functional connectivity of the left/right thalamus to V1 and A1, respectively, is depicted in Fig. 3. During pre and post scans the left and right thalamus exhibited stable temporal correlations of BOLD time series to V1 and A1. During the MMGF-exposure, however, this coupling was progressively reduced.

**Figure 4:**
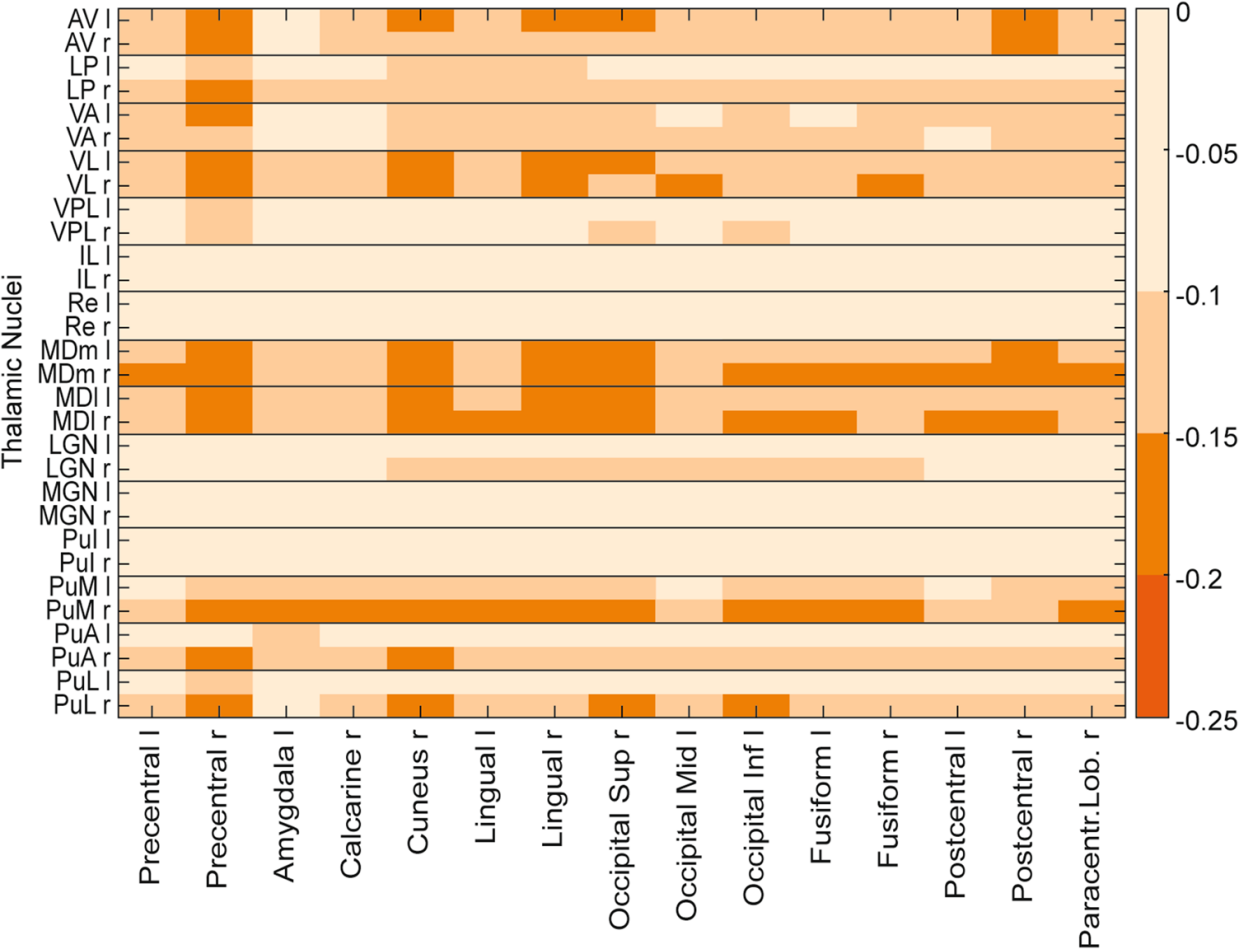
Functional connectivity decreases for thalamic subregions. Correlation decreases between thalamic subregions are displayed in an exploratory analysis. The connectivity matrix includes all thalamic subregions (l: left; r: right) defined in the AAL3 atlas and all cortical regions for which at least one decrease exceeded − 0.15. *AV* anteroventral, *LP* lateral posterior, *VA* ventral anterior, *VL* ventral lateral, *VPL* ventral posterolateral, *IL*: intralaminar, *Re* reuniens, *MDm* mediodorsal medial, *MDl* mediodorsal lateral, *LGN* lateral geniculate, *MGN* medial geniculate, *PuI* pulvinar inferior, *PuM*: pulvinar medial, *PuA* pulvinar anterior, *PuL* pulvinar lateral.

To explore the connectivity profiles of different thalamic subregions, we used the thalamus parcellation of the AAL3 atlas. No Ganzfeld-induced thalamo-cortical correlation increases above 0.1 were present. Figure 4 displays correlation decreases for all thalamic subregions to cortical regions where at least one connection was modulated by at least − 0.15. This exploratory analysis indicates that thalamic decoupling is strongest for ventral lateral, and mediodorsal thalamic nuclei to pre- and postcentral regions, the cuneus, as well as visual processing-related regions e.g., occipital regions and lingual and fusiform gyrus. Furthermore, the pulvinary-visual pathway showed decreased correlation values. Lateral and medial geniculate, as well as intralamiar nucleus and reuniens, showed little modulation.

### MMGF-induced changes in Eigenvector centrality

**Figure 5:**
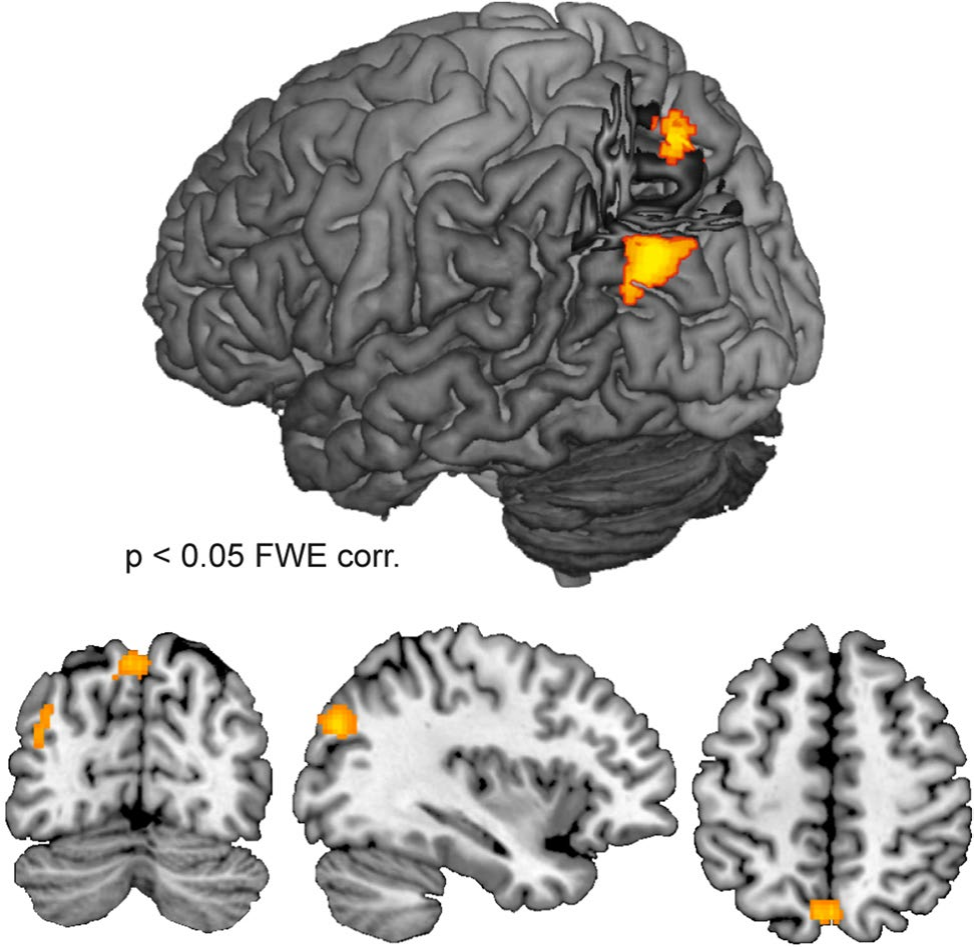
Increases in Eigenvector centrality during MMGF. The assumption-free eigenvector centrality mapping revealed increased centrality of the left inferior parietal lobulus and the precuneus (both core regions of the DMN network) during MMGF when contrasted to averaged pre and post scans.

We used eigenvector centrality mapping, a data-driven analysis tool (without prior assumptions), to test for further changes in resting-state network properties throughout the whole brain. We compared eigenvector centrality maps during MMGF to averaged pre and post scans. This contrast, computed in a second-level ANOVA design, revealed increased centrality (p < 0.05 FWE corrected on the cluster level) in two core regions of the default mode network (DMN), namely the left inferior parietal lobulus (IPL; peak: x = -38, y = − 76, z = 30; cluster size: 192 voxel; T = 5.41) and the precuneus (PRE; peak: x = 0, y =  − 70, z = 54; cluster size: 106 voxel, T = 5.30; Fig. 5).

## Discussion

In the present study, we explored the induction of an ASC with a non-pharmacological method, namely the multimodal Ganzfeld (MMGF), which consists of high intensity exposure to unstructured auditory and visual stimulation. Retrospective questionnaire assessment confirmed that the ASCs did not significantly differ when induced during fMRI scanning or outside the fMRI scanner. In contrast to the effects observed for serotonergic psychedelics, our resting-state functional connectivity analysis revealed that the most dominant effects were found as a reduced coupling between the thalamus and cortical areas, mainly early sensory regions. This thalamo-cortical decoupling was found to progressively develop over time, as revealed by a time resolved connectivity analysis. Additionally, we assessed changes in global brain network properties by applying eigenvector centrality (EC) mapping. We found increased EC within core regions of the default mode network, which could relate to the inward-directed phenomenological nature of the MMGF-induced state.

### Thalamo-cortical coupling and ASCs

Thalamic dysfunction is well known to play an important role for the emergence of different psychopathologic symptoms, particularly with regards to perceptual distortions. Dysfunctional thalamic filtering is reflected by reduced pre-pulse inhibition in schizophrenic patients, which is also used as a measure to validate animal models of schizophrenic symptoms^35,36^. Schizophrenic patients also report an uncontrolled instream of information from the senses, which is experienced as overwhelming^21,22^. Previous pharmacological studies indicate that ASC symptoms could also be driven by reduced sensory filtering of the thalamus, reflected by increased thalamo-cortical coupling^22^. The suggestion that ASC effects directly relate to thalamo-cortical interactions indicates that the thalamus could be affected in the MMGF-induced state as well.

Antithetical to schizophrenia and the findings in psychedelic-induced states, we observed a reduction in thalamo-cortical coupling during MMGF exposure. We present an explorative analysis in terms of whole brain ROI-to-ROI connectivity changes (Fig. 2). We carefully controlled for potential confounding effects of motion by demonstrating that participants did not move more during the MMGF condition than during the pre and post measurements, which could otherwise substantially affect the validity of the presented findings. Relative selectivity of connectivity changes exists between the thalamus and regions that are known to be anatomically connected with the thalamus, i.e. early sensory regions. We further performed a time-resolved analysis to show that the thalamo-cortical effects demonstrate a plausible development together with the progressing depth of the MMGF induced state. This analysis also revealed consistency across the four lateralization combinations of left/right thalamus and sensory regions (see Fig. 3).

Finding thalamo-cortical decoupling is interesting, as on the one hand the eyes and ears are exposed to input with high signal intensity, namely bright light and loud white noise, which should lead to strong signals from the sensory organs to the brain. On the other hand, these sensory signals did not contain structure, such as differences in brightness or color, edges or shapes, or systematic pitch or loudness variations. This lack of structure is highly unnatural and causes a distortion of normal signal processing to a degree that (pseudo-)hallucinations can occur^13^. Whether participants in the current experiment had such experiences is, however, uncertain as we only assessed ASC experiences with a retrospective questionnaire. Hallucinations during MMGF exposure are typically short-lasting events with relatively clear on- and offset^13^. In contrast, the applied Altered States of Consciousness Rating scale asked for overall changes from an average, normal state. We found the ratings on hallucination-related scales (e.g. elementary and complex imagery) to be variable across participants, which is most likely due to the fact that the data do not allow to disambiguate if the general occurrence, or the amount of such events in relation to the whole MMGF period was to be reported. The change in experience that was rated highest was reduced vigilance. Taken together, this suggests that the MMGF-induced state creates a regime of sensory processing in which hallucinatory events are more likely to occur. To directly relate their occurrence with fMRI data, however, a precise tracking of such events in time would be necessary, which remains a task for future research. Interestingly, in contrast to ASCs induced by psychedelic substances, our data do not show increased, unfiltered signal propagation to the cortex.

One previous study explored the ASC described as shamanic trance by measuring changes in resting-state connectivity in brains of shamans who entered a trance state inside the fMRI scanner, guided by rhythmic drumming sound^23^. Similar to our data, they also found a reduction of connectivity from the thalamus to sensory regions, despite the substantial amount of sensory input. Taken together, the reduced thalamo-cortical interaction is not a result of reduced sensory input but can be observed in situations of highly monotonous and unstructured sensory stimulation.

Another situation where changes of thalamo-cortical interaction are well document is sleep^37^. In particular, reduced thalamo-cortical interaction is found as a feature of early sleep states (N1) in resting-state fMRI^38,39^ and intracranial electrophysiological recordings^40^. The MMGF-induced state is often phenomenologically described as a hypnagogic state; i.e. a state between wakefulness and sleep. Indeed, it has been reported that this phenomenological description matches with the finding that the EEG power spectrum of the MMGF state lays in between wakefulness and early sleep states^41^. A direct comparison of the MMGF data with existing sleep data^39,42^ would be informative for identifying resting-state signatures that distinguish the MMGF state from early sleep states.

The applied measure of functional connectivity for fMRI resting-state time series reflects the correlation of BOLD signal between the thalamus and cortical regions. Thereby, it cannot be directly inferred that the thalamus has less influence on the cortex; cortico-thalamic feedback signals could instead be reduced, or both. The main challenge in investigating thalamo-cortical interactions with resting-state functional connectivity is the small size of subthalamic nuclei in combination with the relatively low spatial resolution of full-brain resting-state fMRI scans (e.g. 3 mm^3^ in the study at hand). To the best of our knowledge, until now, no comparison data is available, where fine grained thalamic parcellation were applied in studies where ASCs were investigated. Different connectivity studies that have investigated thalamo-cortical interactions, did not reveal one-to-one mappings of thalamic subregions with cortical regions^43^. Instead, competing thalamo-cortical networks were indicated^44^. Due to these limitations, our analysis of subthalamic connectivity changes remain exploratory. Conversely, this analysis did not display the strongest modulations for sensory thalamic subregions (auditory: MGN, visual: LGN). Instead, anterior and mediodorsal connections to visual, somatosensory and motor regions showed strongest decreases together with aspects of the pulvinary-visual pathway. An explanation for this could be that the character of the MMGF exposure comes with no reduced sensory input but a lack of structure. However, it remains a task for future research to test if these initial hints reflect more complex thalamo-cortical circuitries that drive the observed main effects, potentially via indirect pathways, including top-down cortico-thalamic mechanisms.

In sum, the reduced thalamo-cortical coupling displays a hallmark characteristic of the MMGF induced connectivity state of the brain, while it remains a question for future research to identify the exact thalamo-cortical and cortico-thalamic mechanisms that contribute to specific experiences.

### The default mode network and the MMGF induced ASC

In addition to the hypothesis-driven analysis of thalamo-cortical coupling, we applied eigenvector centrality mapping as a data-driven method to analyze large-scale network effects. Eigenvector centrality mapping favors regions that are connected to regions that are themselves central within the whole-brain network, and thus measures the global centrality of each voxel^33^. Our analysis revealed increased centrality in core regions of the default mode network (DMN), namely in the precuneus and the left inferior parietal lobules. DMN activity is classically associated with inwards directed thoughts, mind wandering and general processes when no task is performed^45–47^. A centrality increase in those regions means that during MMGF exposure they are more strongly integrated within the whole-brain connectome. Thereby, the DMN appears to take a more central role in orchestrating other brain processes^48,49^. Consistently, participants of a previous study had reported increased self-awareness as a characteristic feature of the MMGF induced state^13^, confirming the classical attribution of DMN with self-related processing. Additionally, the ASC of shamanic trance has been characterized as an inward directed state and consistently was associated with increased eigenvector centrality in DMN regions^23^.

In contrast, psychedelic-induced ASCs have been characterized by a functional disintegration of the DMN^4,5,50^. Interestingly, a characteristic feature of LSD and psilocybin-induced states is the reduction of self-importance; reports extend to a full dissolution of the usually-experienced ego^51^. Taken together, our finding of increased centrality can be interpreted to reflect the dissociation from the outer world and more inward-directed processing. This finding is plausible, since opposing effects were found for psychedelic-induced states which are characterized by ego-dissolving effects.

### Imbalance of bottom-up and top-down signaling

Symptoms elicited by different drugs have been discussed as resulting from altered bottom-up and top-down signaling within hierarchical cortical processing^1^. This idea has been fostered within the framework of predictive coding, where top-down signals are thought to reflect predictions about sensory events (i.e. priors) and bottom-up signals comprise sensory signals. Within the cortical hierarchy, predictions are compared and integrated with sensory signals, thus allowing the brain to derive meaning and perceptual interpretations in a fast and efficient way. Imbalance in the interaction of top-down and bottom-up signaling is thought to be a causal mechanism underlying psychotic hallucinations^52^. On the one hand, hallucinations can result from weak or imprecise priors^53^. Alternatively, it is speculated that reduced bottom-up signaling by sensory deprivation can also cause an imbalance leading to hallucinations^1^. The mechanisms of psychedelic-induced hallucinations were also recently discussed in the context of predictive brain processes^54^. In that model, the DMN is considered a top-level source for priors and its functional disintegration reflects a change in top-down influence.

In the case of MMGF, the perceptual deprivation reduced thalamo-cortical coupling, which can be interpreted as reduced bottom-up signaling. In contrast, the top-down propagation of internally generated predictions should be intact. While during normal perception these top-down predictions are compared with structures in the sensory input, during MMGF such integration is not possible due to the lack of structure in the sensory signals. We believe that the maintained top-down predictions can lead to a misallocation of structures into the sensory noise, which can sometimes lead to the experience of hallucinations during MMGF conditions. Our DMN centrality finding even hints at increased influence of top-down predictions during MMGF. Future studies need to address the exact contribution of the DMN to the initiation of priors and top-down signals, as well as on which hierarchical level the assumed misallocation manifests. Our data do not allow to directly link the findings to the potential occurrence of hallucinations, as participants only reported their experiences in retrospect. Future studies should therefore assess in more detail if and when participants experience hallucinations during MMGF exposure to establish direct links with neural mechanisms.

## Conclusion

We found the MMGF-induced state to be associated with a decreased thalamo-cortical coupling and an increase in centrality of the DMN. Taken together, our data suggest that an imbalance of intact top-down signaling in combination with the unnatural reduction of bottom-up input could explain why participants sometimes experience hallucinations during MMGF.
